# A new electromagnetic method for analyzing urban road dust

**DOI:** 10.1038/s41598-026-52446-2

**Published:** 2026-05-14

**Authors:** Grzegorz Tytko, Sylwia Dytłow, Barbara Solecka, Yuedong Xie

**Affiliations:** 1 Faculty of Automatic Control, Electronics and Computer Science, Silesian University of Technology, Akademicka 16, Gliwice, 44-100 Poland; 2 Institute of Geophysics, Polish Academy of Sciences, Ks. Janusza 64, Warsaw, 01-452 Poland; 3 Institute of Physics – Centre for Science and Education, Division of Applied Physics, Silesian University of Technology, Konarskiego 22B, Gliwice, 44–100 Poland; 4 School of Instrumentation and Opto-Electronic Engineering, Beihang University, Beijing, China

**Keywords:** Road dust, Magnetic susceptibility, Urban pollution, Electrical impedance spectroscopy, Coil impedance, Chemistry, Engineering, Environmental sciences, Materials science, Physics

## Abstract

Road dust is a key urban pollutant and indicator of environmental contamination from traffic and industry. Conventional analysis methods are often slow, expensive, and require complex preparation. This study presents a novel electromagnetic method using electrical impedance spectroscopy (EIS) for road dust analysis. The technique leverages the eddy current effect with a specialized measuring coil to determine impedance components from sealed dust samples. Twenty samples from Warsaw, Poland, were analyzed, with results displayed as normalized impedance plots. These plots showed distinct characteristics, enabling clear differentiation of all samples. Magnetic susceptibility measurements confirmed a strong correlation with resistance changes, validating the method’s sensitivity to magnetic mineral content. The results demonstrate that this approach provides a rapid, cost-effective, and non-destructive tool for distinguishing dust samples and assessing magnetic contamination. Furthermore, its electromagnetic nature suggests potential for detecting non-ferromagnetic heavy metals like lead, cadmium, and chromium, opening new avenues for environmental monitoring.

**Supplementary Information:**

The online version contains supplementary material available at 10.1038/s41598-026-52446-2.

## Introduction

**Fig. 1:**
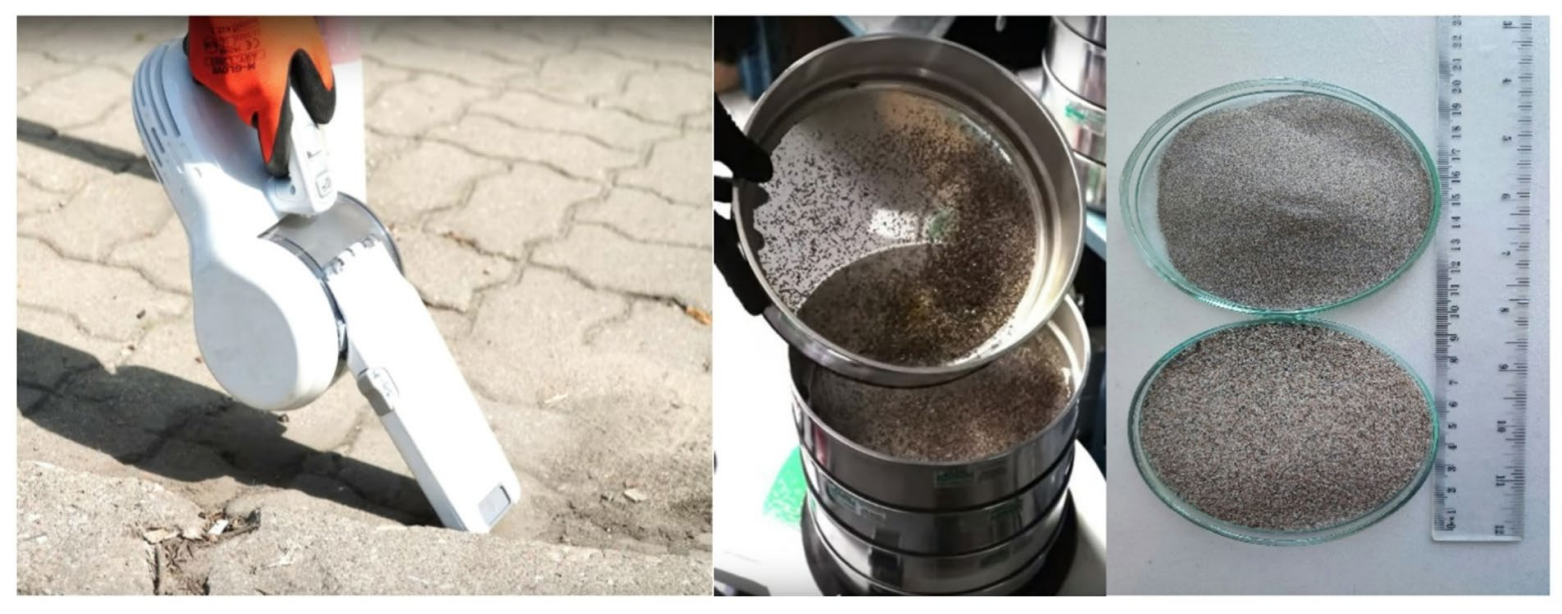
Collection of road dust from a paved street surface covered with cobblestones (left), sieving of the collected material under laboratory conditions to separate it into different granulometric fractions (center), and road dust after sieving, showing distinct grain-size fractions (right).

Road dust is recognized as one of the key components of urban pollution. It is a complex mixture of mineral and soil particles combined with materials of both natural and anthropogenic origin that accumulate on the road surface^1–3^(Fig. 1). The anthropogenic portion mainly derives from traffic-related sources, including fuel combustion, the abrasion of tires and brake pads, and the degradation of road surfaces, along with emissions from industrial activities and low-stack sources^4–9^. It is important to emphasize that the anthropogenic fraction in urban road dust constitutes a complex group of both organic and inorganic pollutants. Among the major inorganic contaminants present in street dust are heavy metals and metalloids (e.g., Pb, Cu, Zn, Cr, Cd, As, Sb, Ni, V), primarily emitted through brake liner attrition, tire wear, and fuel combustion^10^. Furthermore, the dust contains Rare Earth Elements (REEs: La, Ce, Nd) used in modern automotive alloys and specialized glass components^11^. Among the most critical organic pollutants are polycyclic aromatic hydrocarbons (PAHs) such as benzo[a]pyrene and fluoranthene, which are persistent markers of incomplete combustion^12^. From the degradation of the road pavement itself, the dust sequesters bituminous binders composed of high-molecular-weight asphaltenes and maltenes, alongside molecular markers such as hopanes and steranes^13^. Tire wear particles (TWP) contribute styrene-butadiene rubber (SBR) and vulcanization additives. Additionally, road dust is a significant sink for microplastics, specifically tire and road wear particles (TRWP), and black carbon^14^.

The precise assessment of this multi-component mixture requires a sophisticated and costly analytical infrastructure. Inorganic elements are quantified via Inductively Coupled Plasma Mass Spectrometry (ICP-MS)^15^ after microwave-assisted acid digestion^16,17^. Organic speciation requires Accelerated Solvent Extraction (ASE) or Soxhlet extraction, followed by Solid-Phase Extraction (SPE) clean-up and analysis via Gas Chromatography–Mass Spectrometry (GC-MS)^18^. The identification of TRWP and microplastics in the dust matrix is particularly demanding, necessitating Micro-Fourier Transform Infrared Spectroscopy (mFTIR)^19^ for particle identification or Pyrolysis-Gas Chromatography^20^-Mass Spectrometry (Py-GC-MS)^21^ to determine specific polymer mass concentrations, such as the rubber content from tires^22^. Despite the high precision of these conventional environmental assessment techniques, their widespread application is severely hindered by the extreme complexity of sample preparation, which often requires multi-step acid digestion or hazardous solvent extractions. These traditional protocols, involving sophisticated analytical infrastructure like ICP-MS or GC-MS, are characterized by exorbitant costs, low throughput, and a destructive nature that prevents further analysis of the same material. Furthermore, the limited accessibility of these specialized laboratories and the long turnaround times make them impractical for high-density spatial monitoring or rapid screening. Consequently, there is a clear demand for faster and methodologically simpler alternatives that can bridge the gap between expensive, labor-intensive laboratory work and the need for high-resolution urban environmental mapping.

These bottlenecks necessitate the development of proxy methods. While magnetic susceptibility (*χ*) is a proven proxy for ferrimagnetic particles associated with metal^23–38^, it is largely insensitive to diamagnetic or dielectric shifts from non-magnetic polymers, microplastics, or organic binders. We propose Electrical Impedance Spectroscopy (EIS) to bridge this gap, as it probes the complex permittivity of the dust, capturing both metallic/conductive and organic/dielectric shifts.

A wide spectrum of human-induced activities, such as the burning of fossil fuels, industrial manufacturing, vehicle traffic, and the mechanical abrasion of tires, contributes considerably to the emission of magnetic minerals and heavy metals into the environment^23–26^. Magnetic-based approaches have been increasingly employed to trace such contamination, as numerous investigations have confirmed significant links between magnetic parameters and pollutant concentrations, particularly heavy metals. This methodology has been effectively utilized across multiple types of environmental matrices, including soils^27,28,30^, urban road dust^32–34^, sediments^3,36^, vegetation surfaces such as leaves^37^, and suspended particulate matter^38^.

In this study, a measurement approach that meets all these requirements was proposed. For the first time, electrical impedance spectroscopy based on the eddy current phenomenon was applied to the investigation of road dust. A dedicated measurement coil was designed and constructed to achieve the high sensitivity required for this type of analysis. After the coil was supplied with alternating current, road dust samples were placed inside it, and the impedance components were measured. Measurements were performed for 20 samples of different compositions, and the results were presented in the form of impedance diagrams. In all cases, the observed impedance changes allowed for the clear differentiation of individual samples. Subsequently, magnetic susceptibility measurements were carried out as an indicator of the presence of magnetic mineral contaminants in the road dust. The analysis revealed a distinct correlation between the magnetic susceptibility of the samples and the measured resistance values. Therefore, the developed method can be used not only to distinguish between road dust samples but also to estimate the content of magnetic mineral pollutants after establishing an appropriate measurement scale. Importantly, the electromagnetic nature of this method also allows for the inference of heavy metal contamination levels, even for metals that do not exhibit magnetic properties. Numerous advantages, including simple sample preparation, low cost, straightforward interpretation of results, and short measurement time, confirm the considerable potential of the proposed measurement method, which can be applied both for preliminary assessments and for detailed analyses of contamination levels in road dust.

## Study area and methodology

### Study area

All road dust samples were collected in Warsaw, which is the capital of Poland, located in the central part of the Mazovian region along the Vistula River (52°13′48″N, 21°00′40″E). The city extends over 517.24 km² and is situated on the flat Masovian Plain at an average elevation of approximately 100 m above sea level. Warsaw experiences a humid continental climate with distinct seasonal variability. Average winter temperatures range from − 5 °C to 0 °C, whereas in summer they typically vary between 19 °C and 24 °C. Annual precipitation reaches 500–600 mm, with the highest rainfall observed between May and September.

### Characteristics of road dust

Research on road roughness^39^ demonstrates that segments with a high International Roughness Index (IRI > 3.0 m/km) exhibit higher concentrations of wear particles due to increased mechanical degradation of vehicle components, a process that occurs independently of traffic density. Road topography, specifically the slope, further accounts for the observed variability. Downhill segments are prone to higher accumulation of anthropogenic material compared to uphill sections due to intensified braking forces. These forces trigger the mechanical abrasion and friction-induced release of particles from brake pads and discs. Consequently, the moderate nature of the correlations reflects a multifactorial system where traffic volume sets the baseline, but driving behavior (stop-and-go cycles), road deterioration (IRI), and gradient determine the final intensity of the anthropogenic signature in the road dust^39,40^.

The multi-component anthropogenic matrix of Warsaw’s road dust represents a global environmental challenge. The presence of metallic iron, anthropogenic magnetite, heavy metals, PAHs, and microplastics in Warsaw road dust samples^41–44^ aligns with contamination profiles documented across diverse geographical regions.

In Europe and South America (e.g., Spain, Mexico), road dust is consistently characterized by high loads of heavy metals such as Cu, Zn, and Pb from brake wear and tire abrasion, alongside Platinum Group Elements (Pt, Pd, Rh) from catalytic converters^45–47^. In Asia, particularly in megacities across China, India, and South Korea, research has identified extreme enrichment of Fe-bearing minerals, magnetic spherules, and toxic elements like Cadmium (Cd) and Chromium (Cr) originating from both intensive traffic and large-scale industrial activities^48–51^. Similarly, studies in Afghanistan, Taiwan, Bangladesh, and China emphasize the role of organic pollutants (PAHs) and carbonaceous matter derived from vehicle exhaust and low-stack emissions^52–55^.

Furthermore, in regions with arid climates like the Middle East (e.g., Iran, Saudi Arabia, Turkey), the urban dust matrix exhibits unique characteristics due to the interaction between natural geogenic silicate dust and anthropogenic residues from the oil and gas industry, such as Ni, V, and petroleum hydrocarbons^56–58^. Global investigations further highlight microplastics (MPs) as a persistent emerging co-pollutant; studies in Vietnam, Japan, Nepal^59^, Germany^60^, and Australia^61^ confirm that synthetic polymers like polyethylene (PE), polypropylene (PP), and tire wear particles (TWP) are now ubiquitous in street dust, further complicating the urban environmental signal.

For decades, environmental magnetism has been established as a successful international standard for the indirect monitoring of urban pollution. As demonstrated in studies across London, Mexico City, and Shanghai, magnetic parameters serve as a reliable proxy for heavy metal concentrations^62–64^. The long-standing success of these magnetic proxy methods in diverse geographical settings provides a strong precedent for the introduction of other physical indirect techniques. In this context, the results of this study suggest that Electrical Impedance Spectroscopy (EIS) holds similar, if not broader, promise as a complementary proxy tool. While magnetic methods primarily target ferrimagnetic minerals, the EIS signal responds to the total conductive and dielectric load of the anthropogenic matrix, including metallic iron, carbonaceous soot, and microplastics. Therefore, building upon the established legacy of magnetic monitoring, EIS emerges as a promising, universal indirect method for future international environmental assessments.

## Urban road dust sampling strategy

**Fig. 2:**
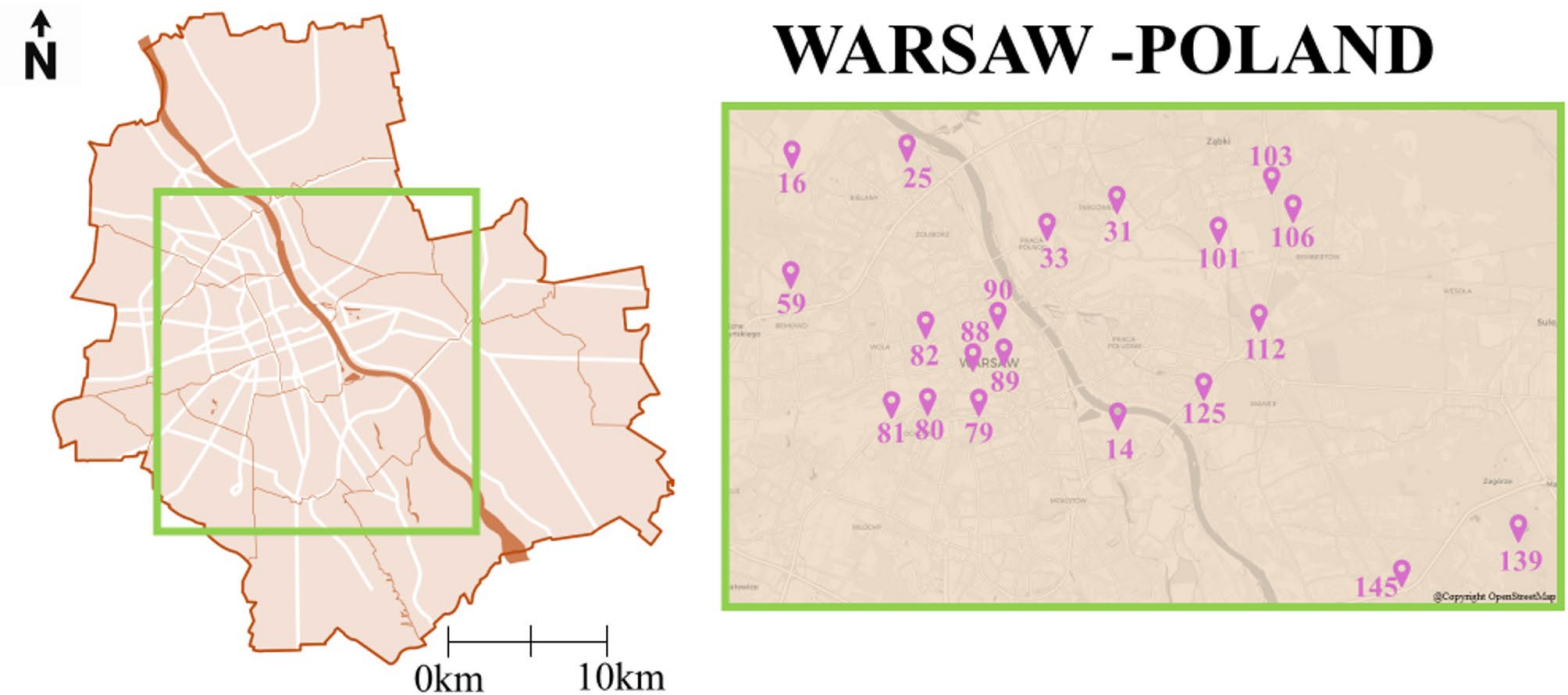
Map of Warsaw, Poland, showing the locations of road dust sampling sites collected during the field campaign. Source of background map: OpenStreetMap contributors (https://www.openstreetmap.org/).

A total of 20 sampling points were selected across the city to capture spatial variability associated with different land use patterns (Tab S2 (Supplementary Materials)) and levels of traffic intensity (Fig. 2, Table S1). Sampling was undertaken from September to November 2023, exclusively on dry days and in accordance with the official street cleaning schedule provided by the Warsaw City Hall^65^. The timing of sampling was carefully adjusted to avoid the immediate aftermath of street sweeping, ensuring that the collected material represented natural surface dust accumulation.

To reduce the potential impact of atmospheric dynamics and long-range pollutant transport, the sampling campaign was conducted during the autumn period, when wind speeds below 2 m/s occur most frequently^66^. Such calm conditions promote the retention of locally emitted particles, thereby improving the representativeness of the samples. Additionally, the complex urban layout of Warsaw alters near-surface airflow; the presence of dense building structures and street canyons can cause wind direction deflections of up to 45°, significantly influencing the spatial distribution and deposition of pollutants^67^.

At each sampling site, road dust was gathered with clean, non-metallic tools such as a plastic vacuum cleaner, brush, and dustpan, ensuring that a minimum of 500 g of material was collected. Each composite sample, consisting of several subsamples, was georeferenced using a handheld GPS device and stored in polyethylene bags until laboratory processing. Following air-drying, the samples were sieved with a laboratory shaker LPzE-2e (MULTISERW-Morek, Poland) equipped with a 1 mm mesh. The data on traffic intensity were sourced from the Warsaw Traffic Survey^68^.

The road dust samples collected in the Warsaw metropolitan area represent a complex anthropogenic matrix, the composition of which has been established in previous research conducted at the same or similar sampling locations. The mineralogical and chemical profile of the dust is characterized by a high concentration of anthropogenic magnetite, primarily in the form of magnetic spherules from combustion processes, and metallic iron (Fe^0^) derived from the mechanical abrasion of vehicle braking systems^44^.

These magnetic phases are accompanied by a significant load of heavy metals, including Fe (with concentrations reaching up to 5%), Zn, Cu, and Pb, as documented in geochemical analyses of the study area^41^. Furthermore, the organic fraction of the dust contains high baseline concentrations of 16 priority Polycyclic Aromatic Hydrocarbons (PAHs), which are strongly correlated with carbonaceous combustion products like soot and exhibit a clear dependence on particle grain size^43^. Recent investigations have also identified microplastics as a persistent co-pollutant within these urban dust fractions, often occurring in association with the magnetic load^42^.

### Electrical impedance spectroscopy

**Fig. 3:**
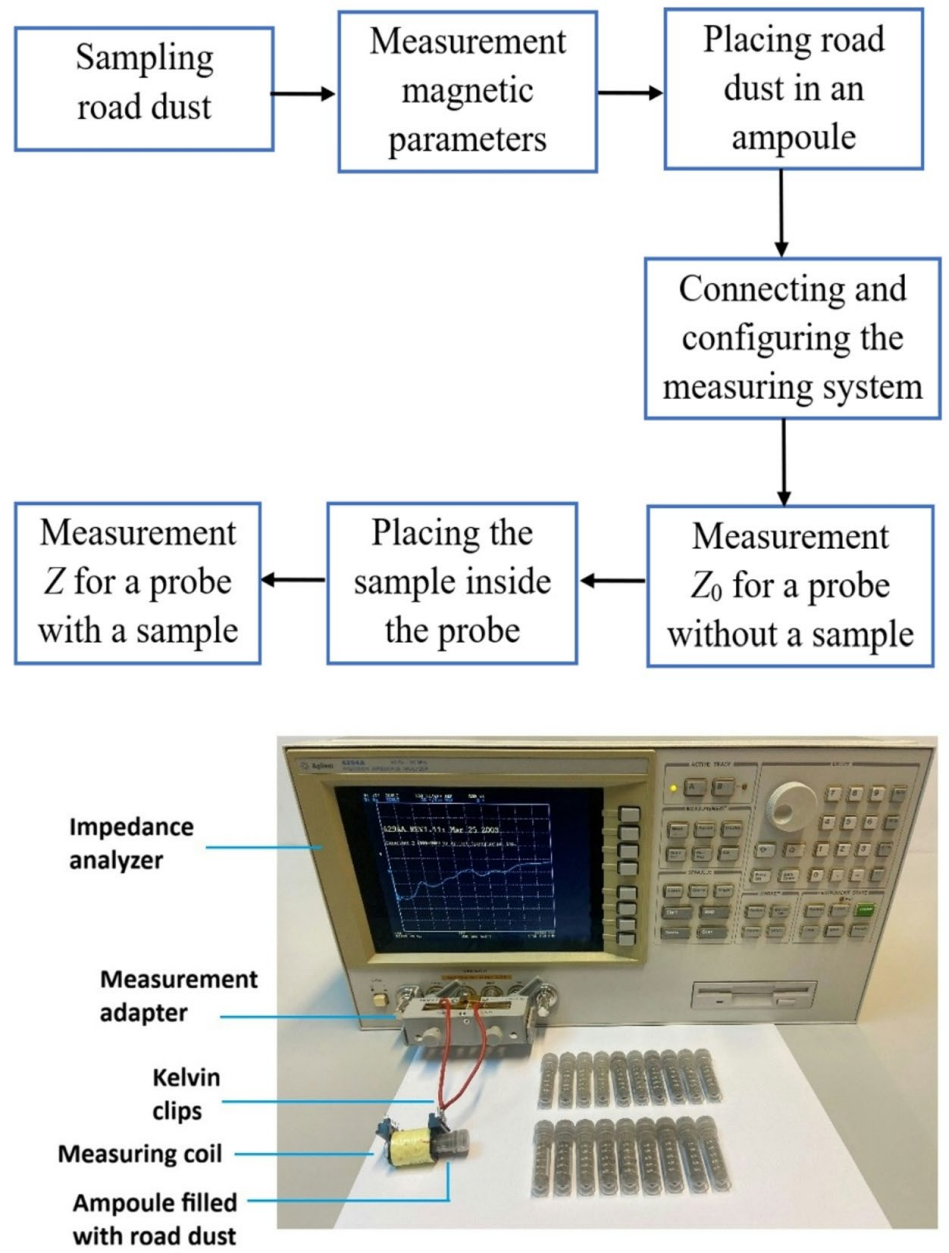
Upper panel: Sketch of the measurement process. Lower panel: Measurement setup comprising an LCR meter, a measuring coil, and ampoules filled with road dust.

The experimental process was conducted in several stages, as shown in the block scheme (Fig. 3). The procedure began with sampling road dust, followed by the determination of its magnetic parameters. The material was then placed in an ampoule, and the measuring system was configured. The final stage involved Electrical Impedance Spectroscopy, consisting of a reference measurement (*Z*_0_​) for the empty probe and the actual measurement (*Z*) for the probe containing the sample.

The measurements described in this study were conducted using an electromagnetic technique in which the shape of the sample affects the measured impedance components of the coil. For this reason, it is essential that all road dust samples maintain the same shape in each measurement series. According to the authors, this requirement has so far represented a major limitation in the use of electromagnetic methods for studying powdered materials. This limitation was overcome by employing identical, hermetically sealed 2 ml ampoules that were completely filled with road dust (Fig. 3). In this way, even when the ampoule is tilted, the powder does not shift, thereby ensuring that each sample retains the same geometry during measurement.

The measurement method proposed in this study is based on the eddy current phenomenon. An eddy current probe powered by alternating current generates a magnetic field. When the probe is brought close to a road dust sample containing electrically conductive components, eddy currents are induced, which affect the impedance of the probe. Variations in the content of conductive components in the road dust lead to changes in the probe impedance. This property enables differentiation of samples with distinct compositions based on the measured impedance values of the coil. Furthermore, by establishing a calibration scale, it becomes possible to determine the content of specific dust components and to identify correlations between selected dust parameters and impedance values.

Eddy current testing allows the differentiation of conductive materials and the determination of their electromagnetic parameters, such as electrical conductivity^69–71^ and magnetic permeability^72,73^. During conventional inspection, the probe^74–79^ is placed in contact with the surface of the tested material. The magnetic flux generated by the probe penetrates the surface and induces currents within the material. However, in the case of powdered materials, such methods are generally ineffective, as the orientation and packing of the grains can influence the measurement results. For this reason, a completely different approach was proposed in this study. For the first time, electrical impedance spectroscopy^80,81^ was applied to the investigation of road dust. This is a volumetric measurement technique in which the sample is placed inside the coil. In this configuration, the magnetic flux generated by the alternating current flowing through the coil fully penetrates the sample, allowing the analysis of materials with different consistencies, including powders.

For the purposes of this study, a dedicated measuring coil was designed and constructed. The coil was wound on a carcass manufactured using 3D printing technology (Fig. 3). Copper wire with a diameter of 0.17 mm was selected. Based on tests conducted by the authors on various coils, it was found that a smaller wire diameter resulted in lower coil sensitivity. Conversely, using a wire diameter greater than 0.17 mm did not result in larger changes in the impedance components. The coil was wound in layers, in a coreless configuration, resulting in a total of 1000 turns. This is a value entirely sufficient for the measurements of the ampoules filled with road dust. The coil was protected against mechanical damage with resin and then wrapped in yellow foil (Fig. 3). The geometric dimensions of the coil were measured using a digital caliper with an accuracy of ± 0.1 mm. The coil length was 25 mm, the inner diameter was 11 mm, and the outer diameter was 18 mm. The coil geometry was adapted to the ampoules used, taking into account the appropriate amount of road dust to ensure reliable and repeatable measurements. The coil inductance *L*_0_, determined in the absence of a dust sample, was 31.2 mH, and the DC resistance DCR = 77 Ω. The self-resonance frequency SRF = 136 kHz was measured, above which the coil’s nature changes to capacitive. The loss angle was also calculated for the coil without a sample. The obtained value *δ* = 0.038 falls within the required range of 0.010 rad to 0.050 rad. Then, the coil was connected with Kelvin clips to the Agilent 4294 A impedance analyzer. Before starting the measurements, a comprehensive fixture compensation process was performed, consisting of three modes. Open compensation made it possible to reduce measurement errors resulting from the influence of fixture stray admittance. In this mode, the clips were open and positioned at the same distance as during the final measurement series. This eliminated parasitic capacitance between the clips, significantly increasing the accuracy of the impedance measurement. The second mode used was short compensation. It involves measuring the analyzer in a shorted state and eliminates residual impedance errors. Using this mode reduces measurement error for low impedance values. Load compensation allows for the reduction of parasitic effects and errors resulting from impedance mismatch. This compensation mode should be performed last and requires the use of a reference in the form of a resistor or capacitor. Precision resistors of 50 Ω or 100 Ω are most commonly used. In the tests described in this paper, such a resistor was used to achieve the best possible impedance matching. For this purpose, an 82 Ω ± 1% resistor was selected, which approximately corresponds to the coil’s resistance. In the first measurement series, preliminary impedance measurements of the coil were performed for four different samples. In the subsequent series, the road dust was removed from the ampoules and refilled in the same manner as in the first series. The measurements were repeated to verify whether the arrangement of particles inside the ampoule affected the results. In both series, very similar impedance values were obtained, differing by no more than 0.2–0.3%.

The impedance of the coil was determined using the following equations for the empty system (*Z*_*0*_) and the sample-filled system (*Z*) :

1 $$\:{Z}_{0}=\:{R}_{0}+j\:{\upomega\:}\:{L}_{0}$$

2 $$\:Z\:=\:R+j\:{\upomega\:}\:L\:\:$$

where the parameters are defined as follows:

*Z*_*0*,_ *Z* – complex impedance of the empty and sample-filled coil [Ω];

*R*_*0*,_ *R*– resistance of the empty and sample-filled coil [Ω];

*j* – imaginary unit;

*ω* – angular frequency (*ω* = 2π*f*, where *f* is the operating frequency) [rad/s];

*L*_*0*_, *L* – inductance of the empty and sample-filled coil [H].

The experimental analysis focused on the changes in these parameters, calculated as Δ*R* = *R* − *R*_0_​ and Δ*L* = *L* − *L*_0_​.

Magnetic susceptibility *χ* describes the degree to which a material becomes magnetized when exposed to an external magnetic field. Its magnitude depends primarily on the concentration, composition, and grain size of magnetic minerals present in the sample^82^. In environmental magnetism, this parameter is widely used as an indicator of anthropogenic magnetic particles, which are often linked to pollution sources emitting ferro- and ferrimagnetic iron oxides and oxyhydroxides.

In this study, air-dried street dust samples were placed in standard 8 cm³ plastic containers *Vo* for magnetic analysis. Measurements were performed using a Kappabridge MFK1-FA (AGICO, Czech Republic), operating at a frequency of 976 Hz. The instrument’s sensitivity of 2 × 10⁻⁸ SI and magnetic field strength of 200 A/m enable reliable detection of low concentrations of ferromagnetic minerals while minimizing the influence of paramagnetic components^82,83^.

Mass-specific magnetic susceptibility *χ* [m³ kg⁻¹] was determined according to Eq. (3):

3 $$\:\chi\:=\frac{K\:\times\:\:V_{0}}{m}$$

where *K* is the volume magnetic susceptibility (dimensionless), *V*₀ is the sample volume [m³], and *m* is the sample mass [kg].

The frequency-dependent magnetic susceptibility *χfd%* was also evaluated to estimate the relative change in susceptibility between low- and high-frequency measurements. It was first determined as the difference between *χlf* and *χhf*^84^, following Eq. (4):

4 $$\:\chi\:fd\mathrm{\%}\:=\:\frac{(\chi\:lf-\chi\:hf)}{\chi\:lf}\times\:100\:\:\:\:\:\:\:\:\:\:\:\:\:\:\:\:\:\:\:\:\:\:\:\:\:\:\:\:\:\:\:\:\:\:\:\:\:\:\:\:\:\:\:\:\:\:\:\:\:\:$$

where *χlf* and *χhf* denote the magnetic susceptibility measured at low and high frequencies, respectively. Traditionally, this parameter is obtained for a frequency ratio of 1:10, as in the Bartington MS2 system (465 Hz and 4.65 kHz)^85^. However, since the MFK1-FA Kappa Bridge operates with a 1:15 frequency ratio (976 Hz and 15.616 kHz), recalculation of *χfd*% values was required. To correct for this difference, the conversion formula proposed by^86^ was applied, as expressed in Eq. (5):

5 $$\:\chi\:fd\mathrm{\%}\left(1:15\right)=\left(\frac{\mathrm{ln}10}{\mathrm{ln}{f}_{m\:HF}-\mathrm{ln}{f}_{m\:LF}}\right)\times\:\:\chi\:fd\mathrm{\%}\left(1:10\right)\:$$

where *χ*_*LF*_ and *χ*_*HF*_ correspond to the low and high measurement frequencies, respectively. Frequency-dependent magnetic susceptibility (*cfd*%) was calculated to assess the relative importance of ferrimagnetic grains close to the superparamagnetic–single domain (SP/SD) threshold. The physical basis of this parameter lies in the magnetic relaxation of fine grains. When the measurement frequency increases, the time available for the magnetic moments of superparamagnetic particles to align with the external field decreases. Particles with relaxation times longer than the period of the high-frequency field become ‘blocked’ and do not contribute to the susceptibility, leading to lower values at higher frequencies^83,86^. According to the interpretative framework of Dearing et al^87^., *cfd%* values are used to estimate the concentration of the SP fraction: values exceeding 4% indicate a significant proportion of SP particles, whereas values below 4% suggest that the samples contain a low percentage of SP particles. In the urban environment, the superparamagnetic (SP) fraction is primarily generated through high-temperature anthropogenic processes, such as vehicle emissions and industrial combustion, which produce fine magnetic spherules. Additionally, significant amounts of SP grains result from mechanical stress and friction-induced oxidation during vehicle braking, where metallic fragments from brake pads and discs are released and subsequently oxidized^88,89^.

The measurements of hysteresis loops were performed using a Vibrating Sample Magnetometer (VSM; Molspin, UK) operating at a maximum magnetic field of 1 T. The obtained loops were corrected for linear paramagnetic contributions before interpretation. From the processed data, key magnetic parameters were determined, including saturation magnetization *Ms*, saturation remanent magnetization *Mrs*, and coercivity *Hc*, representing the total magnetic moment under complete magnetic alignment, the residual magnetization remaining after removal of the external field, and the field strength required to reduce the magnetization to zero, respectively^83^. To determine the remanent coercivity (HCR​), isothermal remanent magnetization (IRM) measurements were complemented by the subsequent application of a stepwise direct-current back-field.

In environmental magnetism, hysteresis parameters provide specific insights into the nature of magnetic carriers. Saturation magnetization (*Ms*​) serves as a proxy for the total concentration of magnetic minerals, while saturation remanence magnetization (*Mrs*​) indicates the ability of these minerals to retain magnetization. Coercivity (*Hc​*) and remanent coercivity (*Hcr*​) measure the ‘magnetic hardness’ and are used to distinguish between different mineralogies, such as low-coercivity magnetite and high-coercivity hematite^82,83,90,91^.

Although these parameters offer individual information, their primary environmental significance lies in their combined interpretation. By calculating the ratios *Mrs​/Ms*​ and *Hcr​/Hc*​, we can utilize the Day-Dunlop^92^ plot to determine the magnetic domain state of the particles. This combined approach is essential for identifying the grain size of the magnetic fraction, allowing us to differentiate between fine-grained, stable single-domain (SD) particles, typical of high-temperature combustion processes, and coarser multi-domain (MD) particles, which often originate from mechanical wear or geogenic sources.

### Statistical data processing and spatial distribution

The Origin2019 software (OriginLab Corporation, US) was utilized to draw all presented graphs and the spatial distribution map. The Delaunay triangulation^93^ scheme implemented into Origin2019 software was applied to interpolate the data between sampling sites on the spatial distribution map.

## Results

**Fig. 4:**
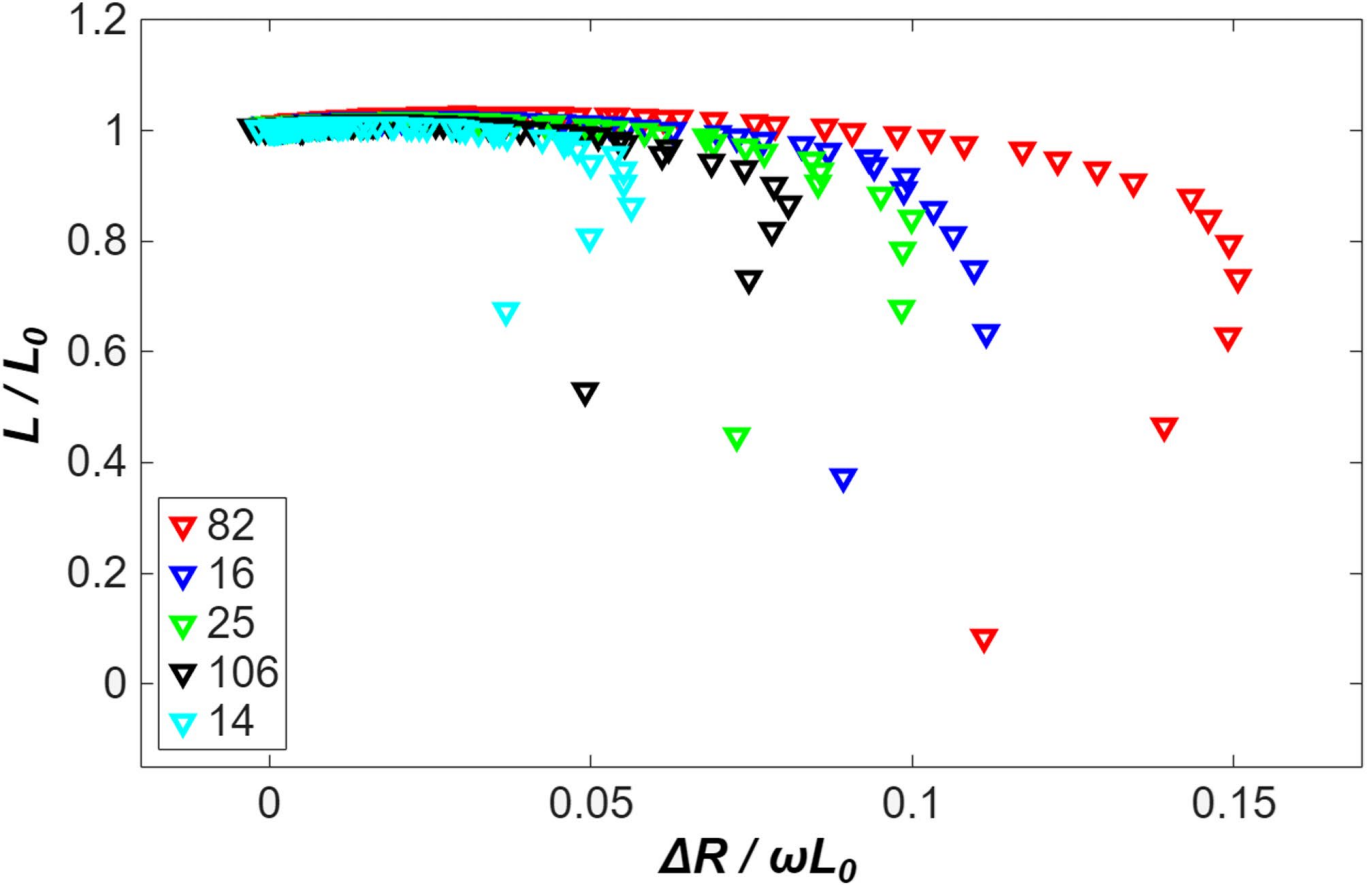
Impedance diagram of the normalized coil impedance components for samples 82, 16, 25, 106, and 14.

**Fig. 5:**
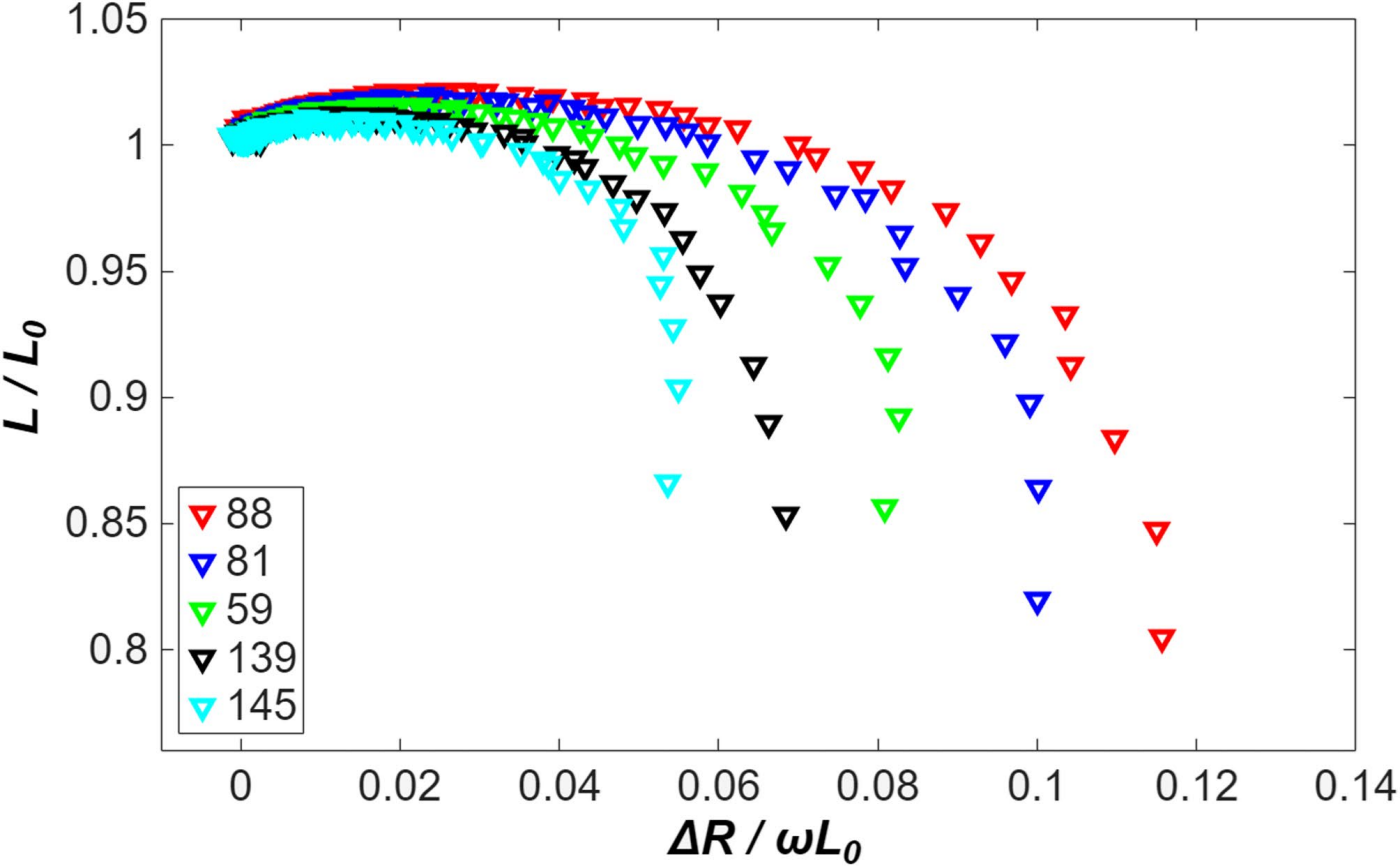
Impedance diagram of the normalized coil impedance components for samples 88, 81, 59, 139, and 145.

**Fig. 6:**
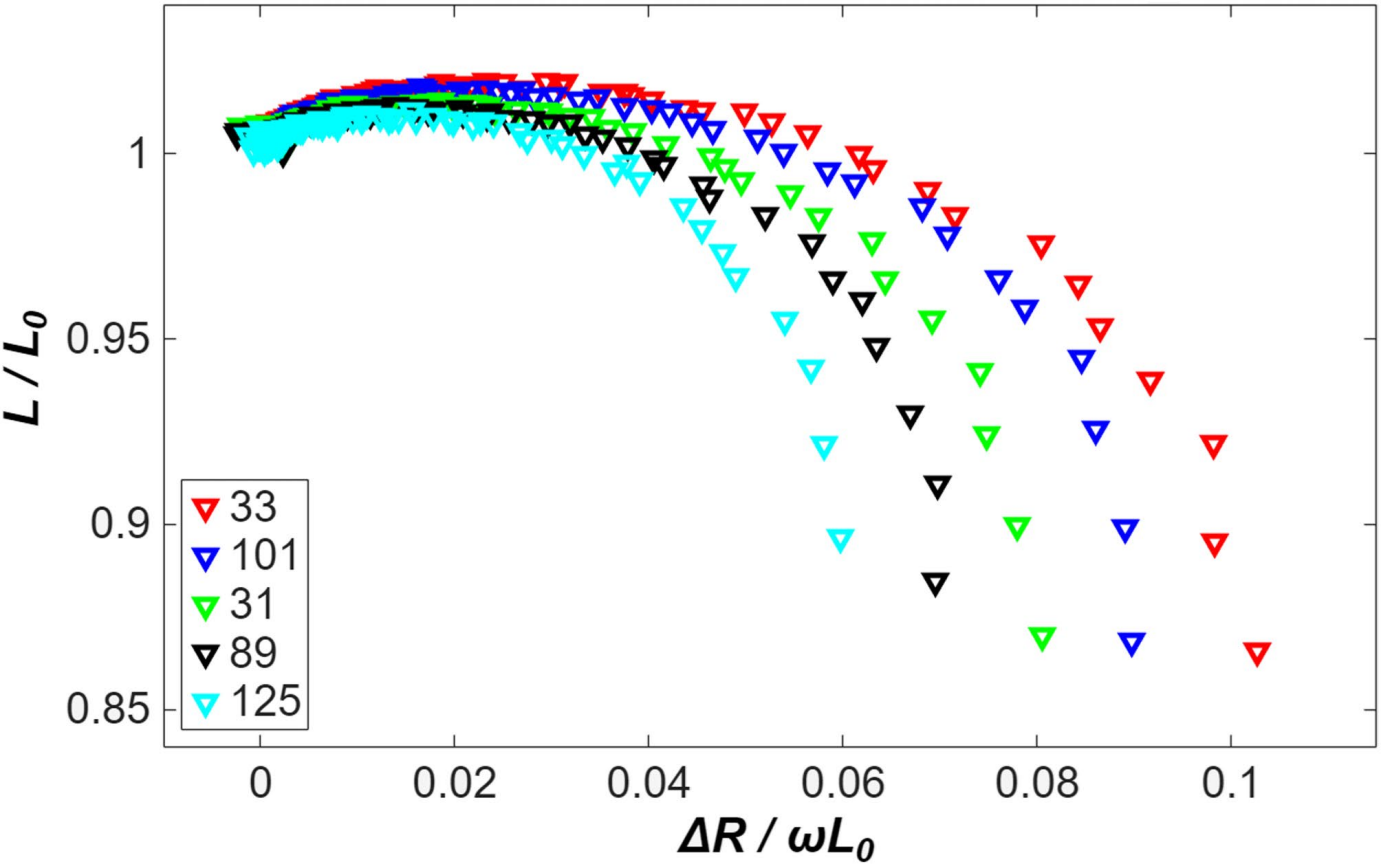
Impedance diagram of the normalized coil impedance components for samples 33, 101, 31, 89, and 125.

**Fig. 7:**
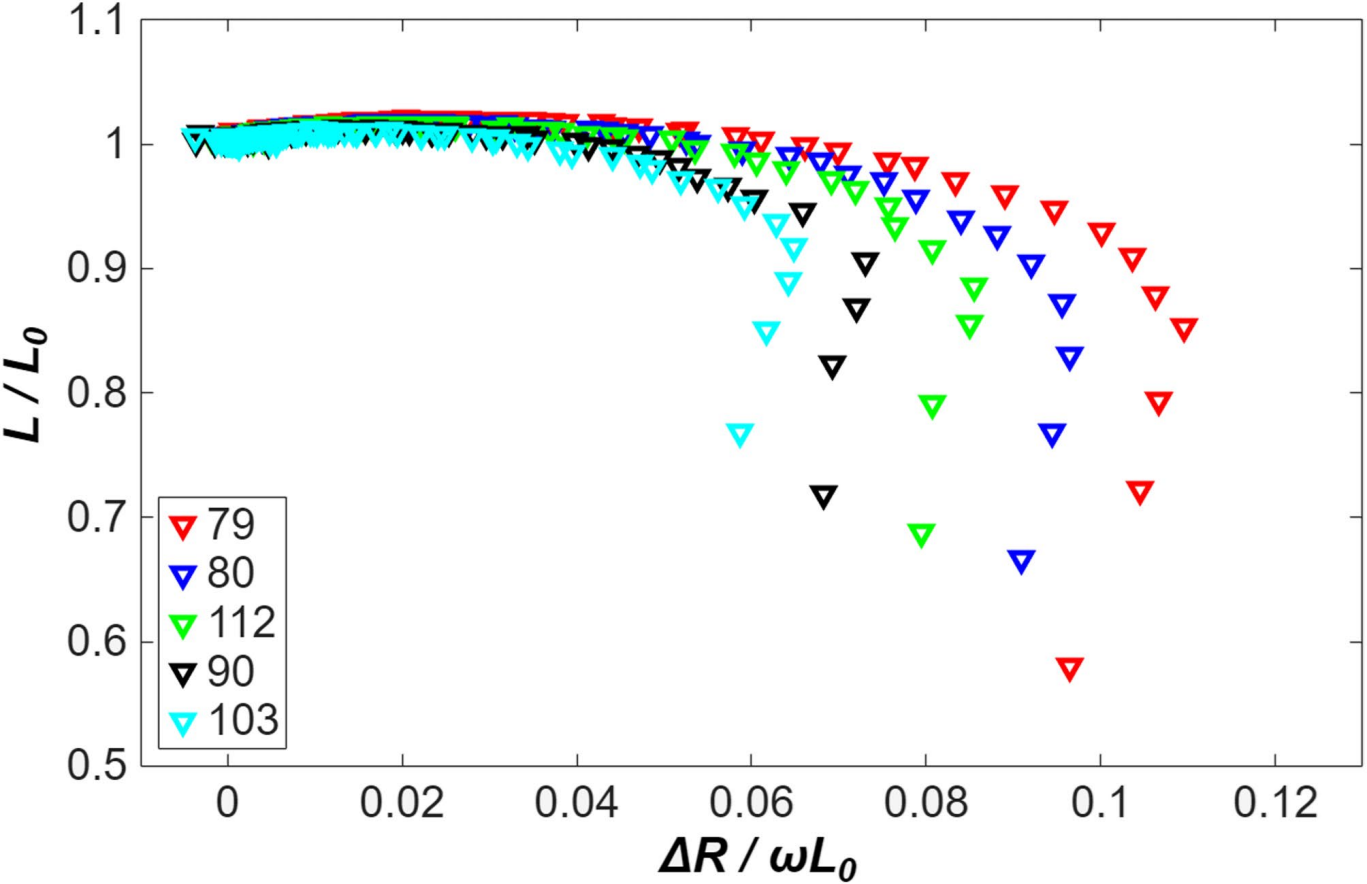
Impedance diagram of the normalized coil impedance components for samples 79, 80, 112, 90, and 103.

The impedance components of the coil were measured using an Agilent 4294 A impedance analyzer with an accuracy of ± 0.08%. In each case, the reported values represent the arithmetic mean of eight individual measurements. The amplitude of the current used was 0.1 mA. A current pulse was chosen because it provided better stabilization of the magnetic field inside the coil than a voltage pulse. Within the frequency range of 100–150 kHz, 200 measurement points were recorded, at 0.25 kHz intervals. In the first step, the impedance of the empty coil was determined as *Z*_0_ = *R*_0_ + *jωL*_0_. Subsequently, a sealed ampoule filled with road dust was placed inside the coil, and the impedance was measured as *Z* = *R* + *jωL*. The analysis considered data obtained for frequencies between 100 kHz and the resonance frequency, approximately 136 kHz. For improved clarity of data presentation, the results were divided into four groups of five samples each, in such a way that they meet the following requirements (Figs. 4, 5, 6 and 7). Each group must contain:

- at least one sample collected on the left bank of the Vistula River and one on the right bank,
- at least 2 samples collected close to each other in order to check whether samples from the same area can be distinguished,
- samples with both low and high magnetic susceptibility values,
- samples with both low and high traffic intensity values.

Meeting these conditions allowed the samples to be grouped while maintaining significant diversity. In the next step, additional requirements were introduced so that samples with similar values for one of the magnetic parameters could also be compared on a single impedance diagram.

e) Similar traffic intensity value (group 1: samples 16 and 14, group 3: samples 33 and 31).
f) Similar saturation magnetization *Ms* value (group 4: samples 112 and 103).
g) Similar, low saturation remanent magnetization *Mrs* value (group 3: samples 101 and 125).
h) Similar, high saturation remanent magnetization *Mrs* value (group 3: samples 33 and 89).

i) Samples with the lowest magnetic susceptibility values (group 2: samples 139 and 145).

j) Samples with very high magnetic susceptibility values (group 2: samples 88 and 81).
k) Similar, high coercivity value (group 4: samples 103 and 112).

In the analyzed frequency range (100–136 kHz), the normalized inductance values (*L*/*L*₀) (Table S3 (Supplementary Materials)) initially increase slightly with frequency, followed by a rapid decrease up to 136 kHz. In the lower frequency range, where *L*/*L*₀ values are the highest, the curves on the diagram are very close to each other, making it impossible to distinguish between the samples based on the obtained results. At higher frequencies, the differences become pronounced, and the curves are clearly separated. Such distinct differences allow for unambiguous differentiation of all samples.

**Fig. 8:**
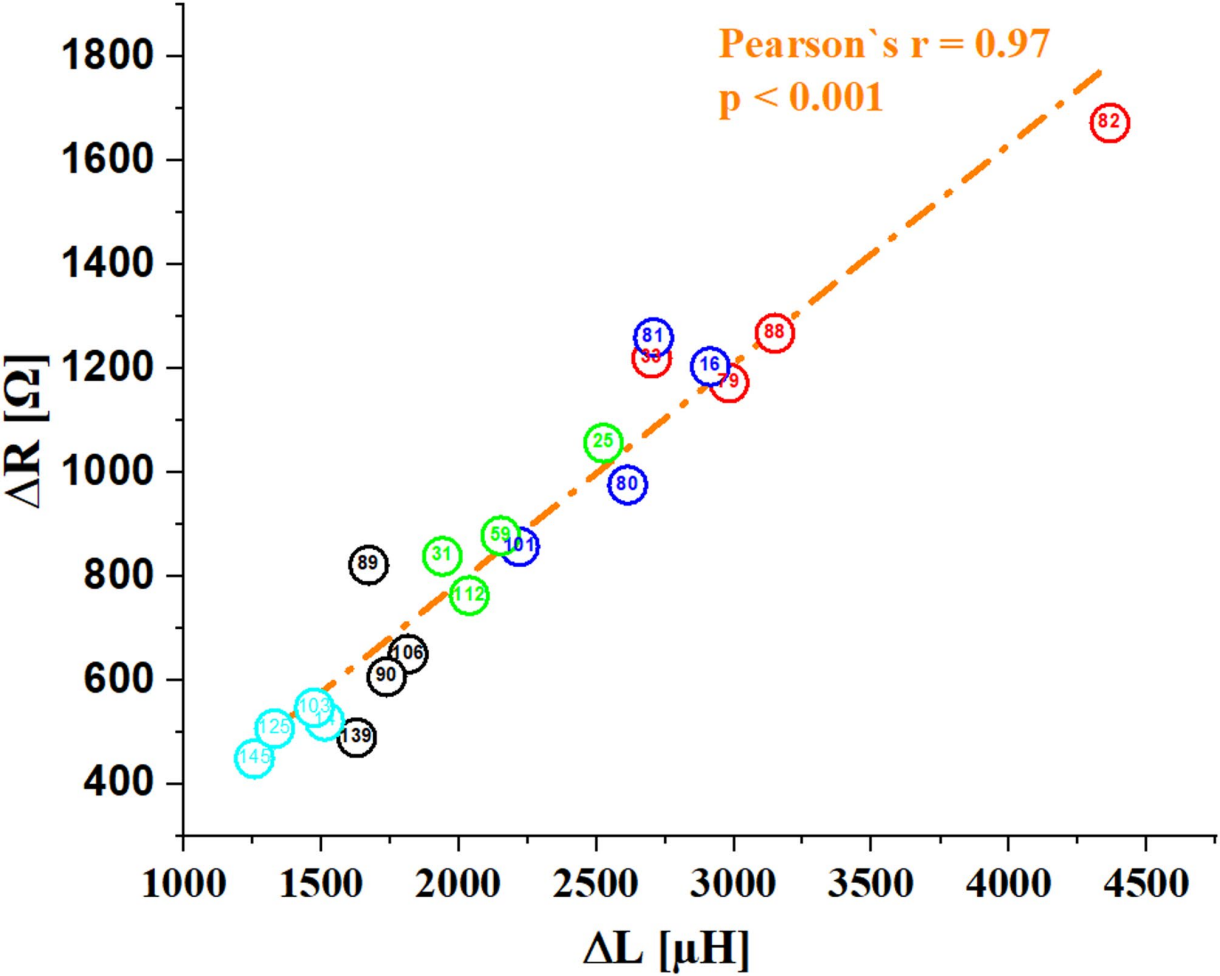
Correlation of Δ*R* and Δ*L values* observed in the experimental samples (*r* = 0.97, *p* < 0.001).

Impedance diagrams are a common form of data presentation in studies employing electrical impedance spectroscopy. Along with techniques that provide data through images or scans, this method is based on direct quantitative values. For quantitative analysis, Δ*R* and Δ*L* values obtained for all samples were determined (Fig. 8). The frequency *f* = 122 kHz was selected, which is approximately 10% lower than the resonance frequency. It was observed that as the coil’s operating frequency increases, so does its sensitivity. The maximum variation in the impedance components was obtained precisely at a frequency of 122 kHz. Further increases in frequency resulted in a decrease in measurement accuracy due to the increasingly strong influence of resonance. The optimal operating frequency of the coil needs to be determined only once, since its value differed only slightly for each sample. This parameter characterizes the coil in question. Thus, replacing the coil with another one necessitates re-determining the optimal operating frequency. If this value is determined incorrectly, the coil’s sensitivity may prove insufficient to distinguish between the samples under test. Numerical data are presented in descending order from the highest to the lowest Δ*R* value. For both resistance and inductance changes, the highest values were obtained for sample 82 and the lowest for sample 145. The differences in these parameters between the two samples amounted to 273% for resistance and 249% for inductance. The high Pearson`s correlation coefficient (*r* = 0.97, *p* < 0.001) further confirms a strong linear interdependence between the resistive and inductive responses across all samples (Fig. 8). This high correlation is physically justified by the fact that any change in the electromagnetic properties of the samples simultaneously modulates the resistive losses and the magnetic permeability, which are the real and imaginary components of the same impedance vector^94^.

## Magnetic properties

### Low-field magnetic susceptibility (*χlf)* and frequency -dependent magnetic susceptibility (*χfd)*

**Fig. 9:**
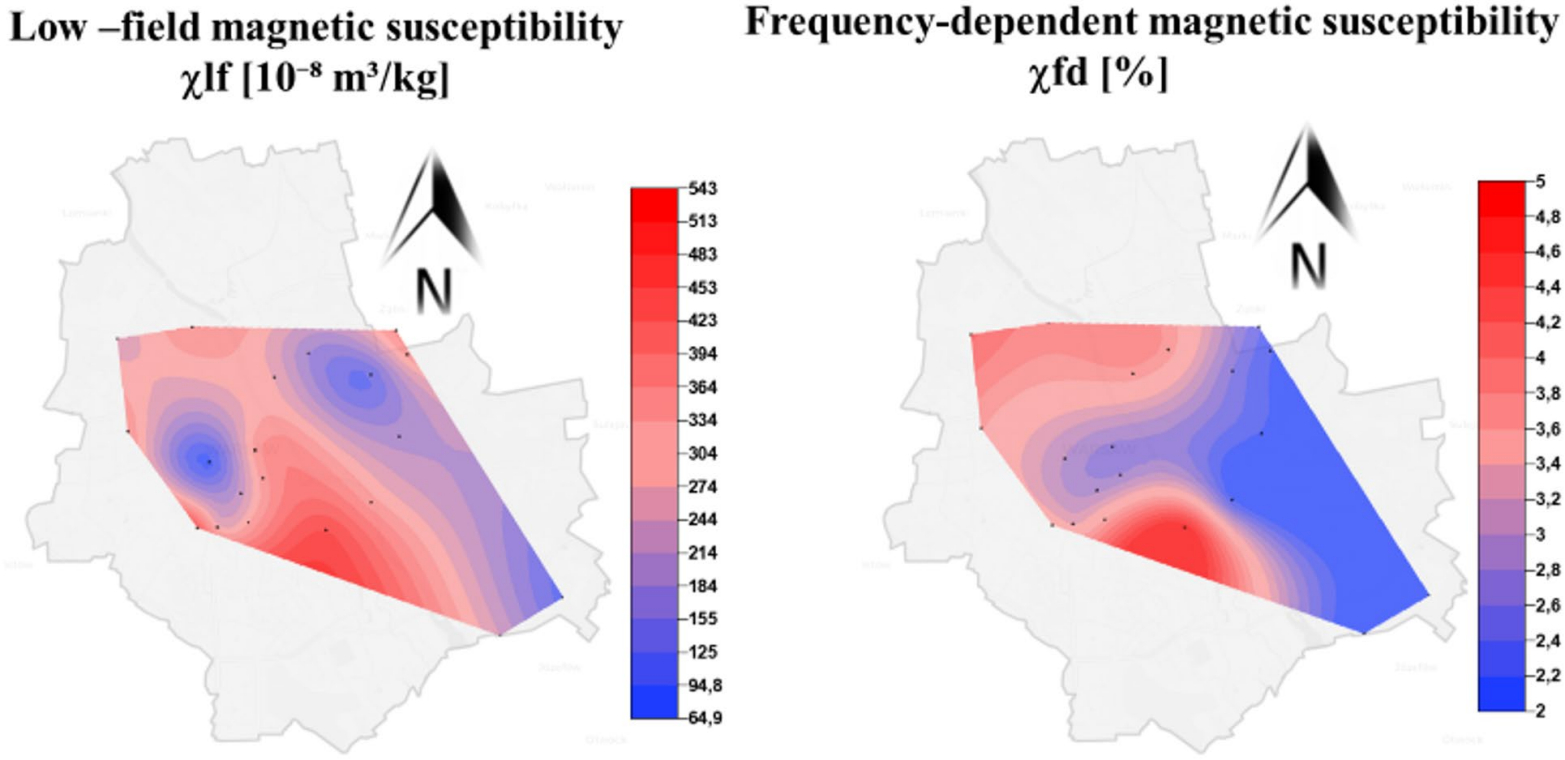
Spatial distribution maps of magnetic parameters in the study area: (left) low-field magnetic susceptibility (*χlf​*) and (right) frequency-dependent magnetic susceptibility (*χfd*). The maps were generated using Origin graph software. Source of background map: OpenStreetMap contributors (https://www.openstreetmap.org/).

The low-field magnetic susceptibility *χlf* (Fig. 9) values of the road dust samples ranged from 39.9 × 10⁻⁸ m³/kg to 545.3 × 10⁻⁸ m³/kg, with a mean value of 238.2 × 10⁻⁸ m³/kg and a standard deviation of 137.6 × 10⁻⁸ m³/kg.

The obtained *χfd* values are relatively low, indicating a limited contribution of superparamagnetic (SP) grains in the analyzed samples. The minimum *χfd* value was 2.1%, while the maximum reached 4.62%, with a mean of 3.4% and a standard deviation of 0.67% (Table S1 (Supplementary Materials)).

The spatial analysis of road dust samples reveals a significant disparity between the left (western) and right (eastern) banks of the Vistula River in Warsaw (Fig. 9). The left-bank districts, characterized by dense urban development and diverse building height profiles, exhibit higher levels of magnetic and electrical loading (Table S1, Table S3). Specifically, hotspots such as sites 82, 88, and 81 show low-field magnetic susceptibility (*χlf*​) values reaching up to 545.3 × 10 − 8 m3/kg and electrical resistance changes (R−R0​) as high as 1672.1Ω. These elevated signals correspond to areas where the urban morphology increases surface roughness, thereby limiting the effective dispersion of traffic-related particulates^95,96^.

Conversely, the right-bank samples (e.g., sites 101, 112, 139) generally show lower magnetic concentrations, often aligning with ‘Open Area’ profiles. The Vistula River valley likely serves as a strategic ventilation corridor, facilitating the removal of pollutants from the eastern part of the city^97,98^. Despite high traffic intensity on major eastern transit routes (e.g., site 103 with 66,897 veh./day), the relatively lower *χlf*​ values suggest that more open spatial arrangements prevent the same level of pollutant accumulation seen in the densely built left-bank center.

### Magnetic hysteresis parameters

**Fig. 10:**
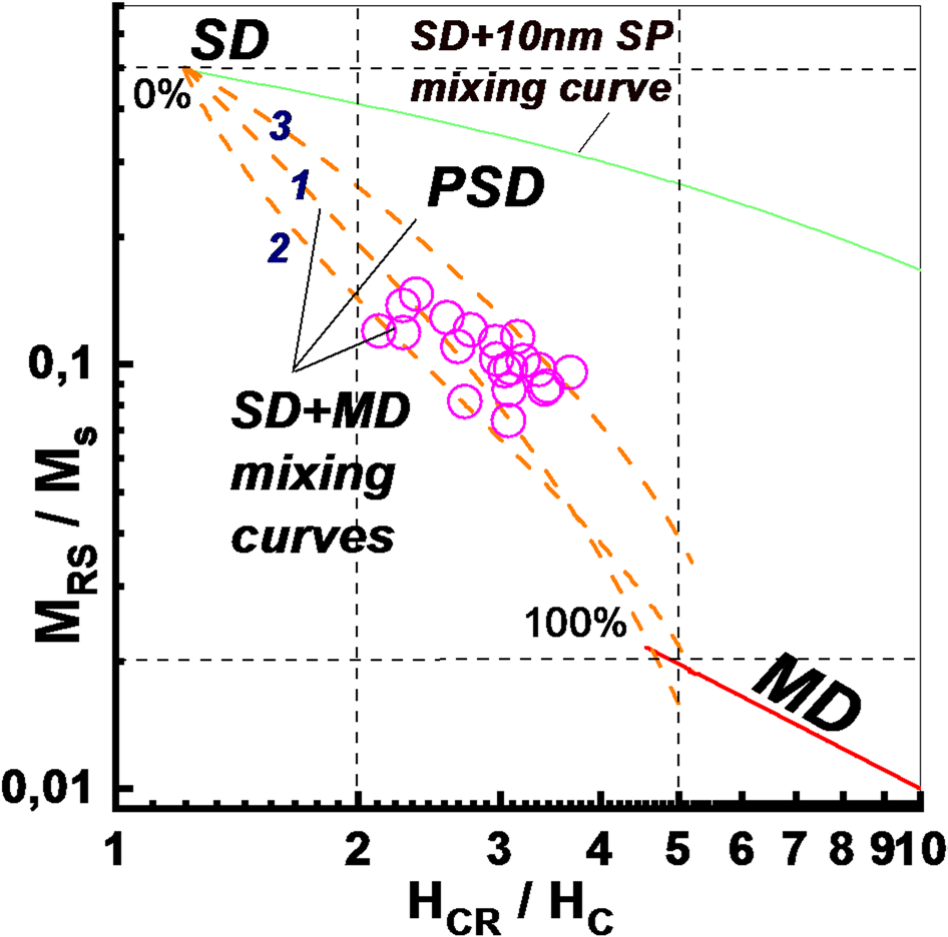
Hysteresis parameter ratios (*M*_*RS*_*​/M*_*S*_​ vs. *H*_*CR*_*​/H*_*C*_​) plotted on the Day-Dunlop diagram^92,99^. The solid red line represents the theoretical trend for multidomain (MD) grains, while the dashed orange lines (1–3) indicate mixing trends between single-domain (SD) and MD grains. The solid green line depicts the calculated mixing curve for SD grains combined with ~ 10 nm superparamagnetic (SP) particles.

The hysteresis ratio values for the analyzed road dust samples exhibit remarkable consistency, with a mean *M*_*RS*_*​/M*_*S*_​ of 0.11 and a mean H_CR_​/H_C_​ ratio of 2.91. As illustrated in the Day-Dunlop plot (Fig. 10, Table S1 (Supplementary Materials)), all data points are tightly clustered within the pseudo-single domain (PSD) region, following the SD + MD mixing curves.

### Comparison between analyzed parameters

**Fig. 11:**
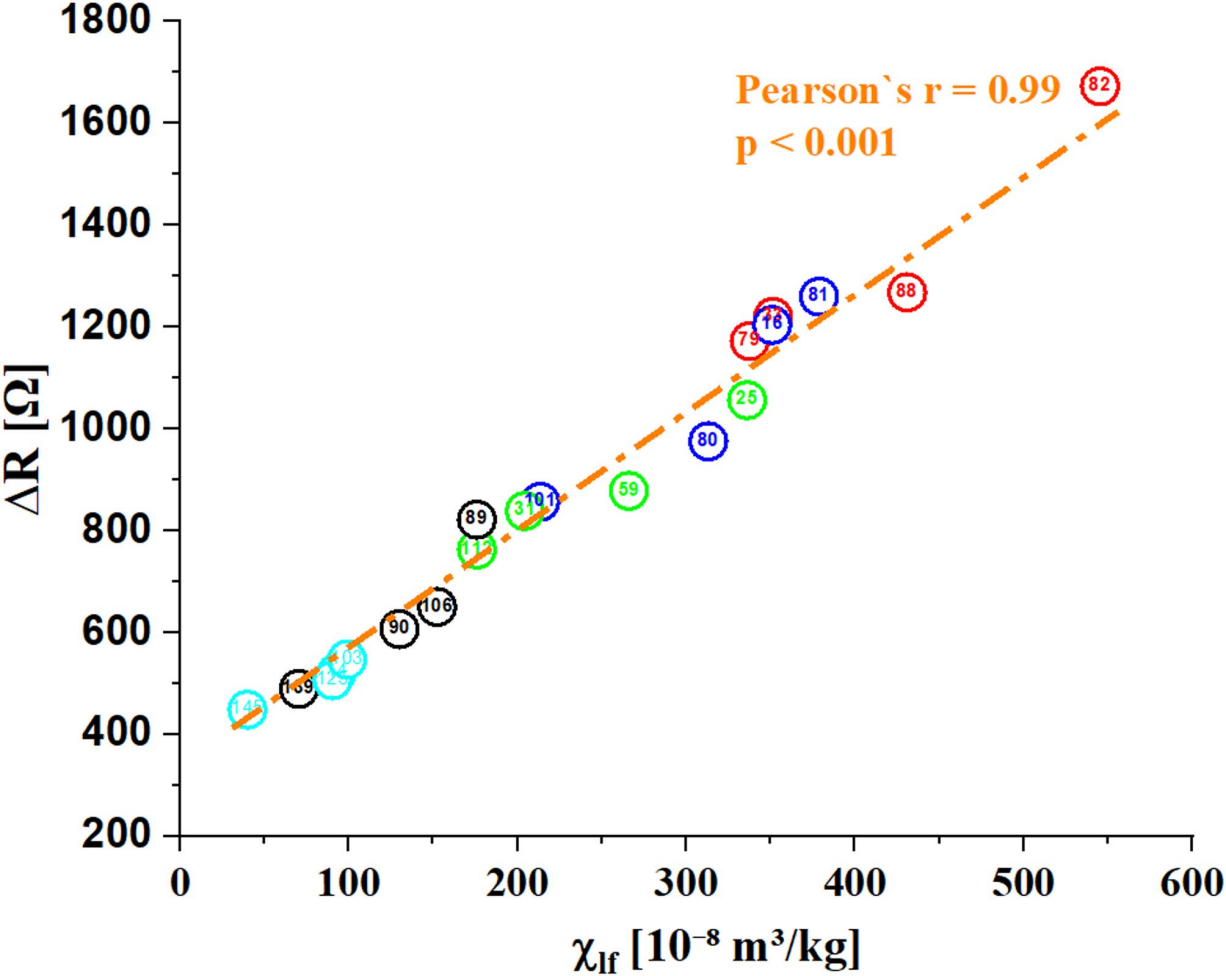
Correlation between low -field magnetic susceptibility (*χlf​*) and Δ*R* for the studied road dust samples.

The magnetic susceptibility values (*χlf*) determined for all samples were compared with the corresponding Δ*R* and Δ*L values*. A very strong correlation was observed between Δ*R* and magnetic susceptibility, as shown in Fig. 11. The regression analysis performed for the 20 road dust samples revealed an exceptionally high Pearson correlation coefficient (*r* = 0.99), with a statistical significance of *p* < 0.001. The samples were sorted by magnetic susceptibility (Table S1 (Supplementary Materials)), from the highest value (545.3 for sample 82) to the lowest (39.9 for sample 145), and are represented by orange bars in the graph. The Δ*R values*, expressed in ohms, are shown as blue bars. The higher the magnetic susceptibility of the road dust sample, the greater Δ*R*. This relationship arises because a higher content of magnetic particles in the dust increases the intensity of the magnetic field generated by eddy currents within the sample. Consequently, resistance changes caused by eddy currents are much larger in samples with a high content of magnetic particles than in those with low magnetic susceptibility.

In the present study, one case confirmed this observation. In the correlation between magnetic susceptibility *χlf* and Δ*R* shown in Fig. 11, a deviation was found for samples 89 and 112. Sample 112 had a *χlf* of 184.5, about 5% higher than that of sample 89 (*χlf* = 176.0). However, the resistance change for sample 112 (Δ*R* = 762.50) was lower than that for sample 89 (Δ*R* = 821.22). To clarify this discrepancy, additional parameters were determined for both samples: frequency-dependent magnetic susceptibility, saturation magnetization, and saturation remanent magnetization (Table 1). The results were conclusive - all three parameters had significantly higher values for sample 89. Therefore, despite its slightly lower magnetic susceptibility, the resistance change for sample 89 was greater than for sample 112.

**Table 1:** Values of the parameters for samples 89 and 112.

Sample ID	ΔR [Ω]	Magnetic susceptibility χlf [10⁻⁸m³/kg]	Frequency dependent magnetic susceptibility χfd [%]	Saturation magnetization Ms [10^− 3^A*m^3^/kg]	Saturation remanent magnetization Mrs [10^− 3^ A*m^3^/kg]
89	821.22	176.0	3.7	296.0	30.2
112	762.50	184.5	2.9	167.8	18.5

Statistical analysis using the Pearson correlation coefficient reveals a moderate positive relationship between traffic intensity and parameters sensitive to the concentration of ferrimagnetic phases. Specifically, traffic intensity correlates with *Ms*​ (0.50), *χlf* (0.48), Δ*R ​* (0.46), *Mrs*​ (0.43), and *χlf* (0.42).

## Discussion

The values of *χlf* in this study are lower than those reported by Dytłow et al.^44^ for street dust from Warsaw, Poland, collected in 2013, where *χ*ranged from 49 to 1025 × 10⁻⁸ m³/kg. In comparison^32^,investigated street dust in Shanghai, China, a city about ten times larger than Warsaw, and reported much higher *χ* values, ranging from 120 to 4040 × 10⁻⁸ m³/kg, with a mean of 810 × 10⁻⁸ m³/kg and a median of 590 × 10⁻⁸ m³/kg. Similar high values were observed in Shanghai by^34^, who found *χ* ranging between 175 and 3367.3 × 10⁻⁸ m³/kg, with a mean of 838.7 × 10⁻⁸ m³/kg. In contrast, studies conducted in the coastal cities of Fujian Province, China - Zhangzhou, Xiamen, and Quanzhou - reported *χ* values from 21 to 911 × 10⁻⁸ m³/kg, with an average of 376 × 10⁻⁸ m³/kg^48^. Even lower values were observed for road dust from Xiamen Island^100^, where *χ* ranged from 25 to 730 × 10⁻⁸ m³/kg, with an average of 250 × 10⁻⁸ m³/kg, which is comparable to the results obtained in the present study.

The interpretation of frequency-dependent susceptibility indicates a low concentration of superparamagnetic (SP) grains in the analyzed samples. According to the criteria established by^87^, *χfd​*values below 4% signify a negligible contribution of the SP fraction to the total magnetic signal^37^. The results for the road dust samples from Warsaw, with a maximum *χfd​* value of 4.62%, confirm that the contribution of superparamagnetic (SP) grains is low. Although the peak value slightly exceeds the 4% threshold, the overall data indicate that the magnetic signal is dominated by coarser grains, with only a minor presence of the SP fraction. This pattern is consistent with previous studies on atmospheric particles^101^, road-deposited sediments^63^, and street dust^32^, where low *χfd* values were also interpreted as evidence of a relatively small contribution from combustion-derived superparamagnetic magnetic grains.

Based on the Day-Dunlop plot generated for the road dust samples from Warsaw, it was determined that the magnetic domain state is dominated by Pseudo-Single Domain (PSD) grains. Similar findings have been reported in studies of road dust internationally. Specifically, research conducted on urban dust in China has also identified the dominance of the pseudo-single domain (PSD) grains as the primary magnetic carrier^32^^48^^64^,,. Overall, such results suggest that the magnetic signal is dominated by coarser, pseudo-single-domain ferrimagnetic particles, rather than by superparamagnetic grains.

The findings of this study demonstrate that Electrical Impedance Spectroscopy (EIS) offers a significant advancement in the environmental assessment of road dust. The distinct separation of curves in the impedance diagrams (Figs. 4, 5, 6 and 7) confirms that each sample possesses unique electromagnetic properties. It was observed that higher concentrations of magnetic particles and heavy metals directly correlate with greater changes in both resistance (*ΔR*) and inductance (*ΔL*). While the inductive response (*ΔL*) was less sensitive—the resistive response (*ΔR*) showed an exceptionally strong correlation (*r* = 0.99, *p* < 0.001) with magnetic susceptibility (χlf​).

A key scientific contribution of this work is the realization that EIS captures a broader physicochemical spectrum than traditional magnetic screening. Because the coil impedance depends on both magnetic and electrical properties, this technique enables the detection of non-ferromagnetic heavy metals—such as lead, cadmium, or chromium, which are electrically conductive. This was clearly illustrated in the comparison of samples 89 and 112 (Table 1); despite having similar χlf​ values, their differing Ms​ and Mrs​ levels were accurately reflected in their distinct impedance signatures. This proves that ΔR provides a more comprehensive “fingerprint” of urban pollutants by integrating magnetization and metallic content.

From a geo-environmental and civil engineering perspective, the moderate correlations (0.4–0.5) between traffic intensity and physical parameters suggest that pollutant accumulation is not a simple linear function of vehicle volume. Instead, the results align with the driving pattern theories of Pandian et al^40^., where emissions at intersections are primarily driven by acceleration, deceleration, and idling. The spatial analysis (Fig. 9) further supports this, showing that high-load “hotspots” are moderated by urban morphology and topography. For instance, the densely developed left-bank districts showed higher accumulation, while the Vistula River valley appears to act as a strategic ventilation corridor that facilitates pollutant removal on the right bank. These findings provide crucial evidence for policymakers, suggesting that urban environmental strategies should prioritize traffic flow optimization and the identification of deposition “hotspots” rather than focusing solely on traffic reduction.

Finally, the proposed methodology addresses the urgent need for cost-effective urban monitoring. Unlike conventional techniques like ICP-MS or GC-MS, which involve high costs, complex acid digestion, and logistical barriers, the EIS-based approach offers:

- Rapid screening: Results for a single sample are obtained within 10 s (narrow frequency band, few measurement points) or half a minute (wide frequency band, many measurement points), enabling high-resolution urban mapping.
- Low operational costs: No consumables or specialized laboratory infrastructure are required.

Non-destructive testing: Samples remain intact for subsequent analyses.

By establishing this accessible framework, this study provides a viable tool for the international scientific community to identify contaminated samples and monitor urban pollution in a logistically and financially sustainable manner.

## Conclusions

The results demonstrate that the application of Electrical Impedance Spectroscopy (EIS) and the measurement of magnetic properties, specifically mass-specific magnetic susceptibility (*χ*), provide a rapid and non-destructive way to characterize urban road dust. The normalized impedance plots effectively differentiate dust samples based on their physical and magnetic parameters. This approach serves as an efficient screening tool for identifying spatial variations and hotspots in urban environments, supporting faster and more cost-effective environmental assessment.

### Limitations and future Work

A primary limitation of this study is the sample size (*n* = 20), which was intended as a proof-of-concept. While current EIS and magnetic parameters effectively differentiate samples, they do not yet provide specific chemical concentrations. To address this, future research will focus on expanding the dataset to include diverse international urban settings across different geographical and climatic regions. This expansion is crucial, as road dust remains a critical global pollutant, and the relevance of the proposed methodology must extend beyond the local context of Warsaw.

A larger, global dataset will enable the development of universal calibration models to correlate impedance signatures and magnetic properties with specific contaminant levels, thereby increasing the method’s utility for the international scientific community. Furthermore, we plan to leverage the electromagnetic nature of Electrical Impedance Spectroscopy (EIS) to examine not only ferromagnetic particles but also non-ferromagnetic components in road dust that conduct electric current. This capability opens new avenues for detecting and quantifying heavy metals such as lead, cadmium, and chromium.

By integrating these physical measurements with chemical analysis, we aim to offer a viable alternative to conventional techniques like ICP-MS or GC-MS. While precise, these traditional methods face significant logistical barriers, including high operational costs, lengthy sample preparation (e.g., acid digestion), and limited laboratory accessibility. In contrast, the proposed methodology seeks to establish a more accessible and cost-effective framework for monitoring urban pollution on a global scale.

### Authors contribution

Conceptualization: G.T. and S.D.; Data curation: G.T., S.D., B.S.; Formal analysis: G.T., S.D., B.S.; Funding acquisition: S.D.; Investigation: S.D., B.S., Y.X.; Methodology: S.D., G.T., B.S.; Project administration: G.T., S.D.; Software: G.T., S.D.; B.S.; Supervision: S.D.; Validation: S.D., Y.X.; Visualization: G.T., S.D., B.S.; Writing – original draft: G.T., S.D.; Writing – review & editing: S.D., Y.X.; The response to reviewers comments and changes in manuscript during revision: S.D., G.T.

## Electronic Supplementary Material

Below is the link to the electronic supplementary material.

Supplementary Material 1

## Data Availability

All data generated or analysed during this study are included in this published article and its Supplementary Materials file.
