# Supplementary material for: A new electromagnetic method for analyzing urban road dust
Source: Sci Rep. 2026 May 14;16:22111. doi: 10.1038/s41598-026-52446-2 (PMC13370015; doi:10.1038/s41598-026-52446-2)
Supplement: Supplementary file 1 — Supplementary Material 1 [file 41598_2026_52446_MOESM1_ESM.docx]

**Supplementary Materials**

**A new electromagnetic method for analyzing urban road dust**

Grzegorz Tytko ^1^. Sylwia Dytłow ^2.*^. Barbara Solecka ^3^. Yuedong Xie ^4^

^1^ Faculty of Automatic Control. Electronics and Computer Science. The Silesian University of Technology. Akademicka 16. 44-100 Gliwice. Poland

^2^ Institute of Geophysics. Polish Academy of Sciences. Ks. Janusza 64. 01-452 Warsaw. Poland

^3^ Institute of Physics – Centre for Science and Education. Division of Applied Physics. The Silesian University of Technology. Konarskiego 22B. 44–100 Gliwice. Poland

^4^ School of Instrumentation and Opto-Electronic Engineering. Beihang University. Beijing. China

**Corresponding author:**

*E-mail address of the corresponding author: Sylwia Dytłow [skdytlow@igf.edu.pl](mailto:skdytlow@igf.edu.pl)

| Sample/  Parameter | Low-field magnetic  susceptibility Xlf [10⁻⁸ m³/kg] | Frequency- dependent magnetic susceptibility Xfd% [%] | Saturation magnetization Ms  [10^-3^ A*m^3^/kg] | Saturation remanent magnetization Mrs  [10^-3^ A*m^3^/kg] | Coercivity [mT] | Remanent coercivity [mT] | R-Ro [Ω] | Traffic intensity [veh./day] |
| --- | --- | --- | --- | --- | --- | --- | --- | --- |
| 82 | 545.3 | 4.6 | 315.7 | 36.7 | 9.7 | 30.6 | 1672.1 | 34575 |
| 88 | 430.9 | 3.3 | 408.1 | 39.1 | 10.1 | 37.1 | 1267.4 | 66900 |
| 81 | 378.7 | 3.8 | 274.7 | 23.9 | 7.5 | 23.0 | 1259.1 | 88910 |
| 33 | 351.5 | 2.8 | 308.3 | 30.0 | 9.6 | 32.2 | 1219.3 | 14900 |
| 16 | 350.9 | 3.4 | 28.4 | 2.8 | 9.4 | 29.1 | 1203.6 | 12002 |
| 79 | 337.7 | 3.8 | 306.2 | 39.5 | 8.7 | 22.5 | 1171.9 | 52292 |
| 25 | 336.2 | 3.6 | 441.6 | 53.3 | 10.0 | 27.6 | 1056.1 | 9900 |
| 80 | 313.0 | 4.5 | 223.0 | 19.5 | 7.7 | 26.1 | 975.1 | 46280 |
| 59 | 266.0 | 3.4 | 198.0 | 17.6 | 8.2 | 28.1 | 877.3 | 25626 |
| 101 | 213.5 | 2.1 | 40.3 | 3.0 | 7.0 | 21.5 | 856.4 | 9900 |
| 31 | 204.4 | 3.1 | 125.5 | 12.1 | 8.5 | 25.7 | 838.0 | 14900 |
| 112 | 184.5 | 3.7 | 167.8 | 18.5 | 9.7 | 25.7 | 762.5 | 11259 |
| 89 | 176.0 | 3.7 | 296.0 | 30.2 | 8.7 | 27.8 | 821.2 | 6390 |
| 106 | 152.3 | 2.4 | 26.9 | 3.7 | 10.2 | 23.2 | 649.5 | 2515 |
| 90 | 130.0 | 3.9 | 80.0 | 6.5 | 6.3 | 17.2 | 606.0 | 1990 |
| 103 | 99.0 | 3.7 | 164.0 | 19.5 | 9.9 | 22.6 | 546.4 | 66897 |
| 14 | 93.0 | 2.9 | 72.0 | 8.1 | 10.4 | 30.7 | 520.5 | 12500 |
| 125 | 90.3 | 3.7 | 43.9 | 4.5 | 8.6 | 25.6 | 506.2 | 4900 |
| 139 | 70.4 | 2.3 | 37.2 | 5.4 | 10.3 | 24.2 | 488.7 | 1990 |
| 145 | 39.9 | 3.2 | 31.4 | 3.8 | 8.0 | 17.0 | 448.3 | 13980 |
| **Min.** | **39.9** | **2.1** | **26.9** | **2.8** | **6.3** | **17.0** | **448.3** | **1990** |
| **Max.** | **545.3** | **4.6** | **441.6** | **53.3** | **10.4** | **37.1** | **1672.1** | **88910** |
| **Mean** | **238.2** | **3.4** | **179.4** | **18.9** | **8.9** | **25.9** | **887.3** | **24930** |
| **SD** | **137.6** | **0.7** | **135.3** | **15.0** | **1.2** | **4.8** | **335.0** | **25645** |

Table S1. The value and statistical parameters for resistance *R-R*₀. magnetic susceptibility *χ*. frequency-dependent magnetic susceptibility Xfd. and traffic intensity of 20 road dust samples.

| **Sample ID** | **Building Height Profile Along the Road (radius 200m from the sampling point)** | **Description of land use category** |
| --- | --- | --- |
| 82 | Low-Rise | Residential area and traffic zone |
| 88 | High-Rise | City Center |
| 81 | Low- Rise (only few buildings) | Commercial area |
| 33 | Mid-Rise | Green area and traffic zone |
| 16 | Low-Rise | Residential area and green area |
| 79 | Mid-Rise | Traffic zone |
| 25 | High-Rise | Residential area and green area |
| 80 | Mid-Rise | Traffic zone and residential area |
| 59 | Mid-Rise | Traffic zone and residential area |
| 101 | Open Area | Green area |
| 31 | Open Area (only few buildings) | Commercial area |
| 112 | High-Rise | Residential and green area |
| 89 | Mid-Rise | City center |
| 106 | High-Rise | Green area and few high buildings |
| 90 | Mid-Rise | City center |
| 103 | High-Rise | Residential area and green area |
| 14 | Mid-Rise | Residential area with high buildings |
| 125 | High-Rise | Residential area with low buildings |
| 139 | High-Rise | Residential area and green area |
| 145 | High-Rise | Traffic zone and residential area |

Table S2. Detailed site characteristics: Building height profiles and land use categories for the studied sampling points. Footnote: Open Area: undeveloped or green spaces with no buildings or single-story structures (0–1 floor). Low-Rise: residential or commercial buildings typically up to 3 floors. Mid-Rise: urban structures typically ranging from 4 to 9 floors. High-Rise: tall buildings or skyscrapers with 10 floors or more. Building Height Profile: defined based on the dominant structure type within a 200m radius of the sampling point to account for local aerodynamic conditions.

| Sample ID | *R*-*R*_0_  [Ω] | *L*-*L*_0_  [µH] | Sample ID | *R*-*R*_0_  [Ω] | *L*-*L*_0_  [µH] |
| --- | --- | --- | --- | --- | --- |
| 82 | 1672.10 ± 1.34 | 4366 ± 3.49 | 31 | 838.00 ± 0.67 | 1941 ± 1.55 |
| 88 | 1267.37 ± 1.01 | 3149 ± 2.52 | 89 | 821.22 ± 0.66 | 1672 ± 1.34 |
| 81 | 1259.06 ± 1.01 | 2708 ± 2.17 | 112 | 762.50 ± 0.61 | 2038 ± 1.63 |
| 33 | 1219.25 ± 0.98 | 2701 ± 2.16 | 106 | 649.51 ± 0.52 | 1816 ± 1.45 |
| 16 | 1203.55 ± 0.96 | 2914 ± 2.33 | 90 | 606.04 ± 0.48 | 1738 ± 1.39 |
| 79 | 1171.87 ± 0.94 | 2982 ± 2.39 | 103 | 546.39 ± 0.44 | 1475 ± 1.18 |
| 25 | 1056.11 ± 0.84 | 2526 ± 2.02 | 14 | 520.47 ± 0.42 | 1513 ± 1.21 |
| 80 | 975.09 ± 0.78 | 2614 ± 2.09 | 125 | 506.15 ± 0.40 | 1331 ± 1.06 |
| 59 | 877.29 ± 0.70 | 2153 ± 1.72 | 139 | 488.72 ± 0.39 | 1629 ± 1.30 |
| 101 | 856.41 ± 0.69 | 2220 ± 1.78 | 145 | 448.34 ± 0.36 | 1257 ± 1.01 |

Table S3. Changes in coil resistance (*R*-*R*₀) and inductance (*L-L*₀) obtained for the tested samples.
